# Associations between television viewing and physical activity and low back pain in community-based adults: A cohort study

**DOI:** 10.1097/MD.0000000000003963

**Published:** 2016-06-24

**Authors:** Sultana Monira Hussain, Donna M. Urquhart, Yuanyuan Wang, David Dunstan, Jonathan E. Shaw, Dianna J. Magliano, Anita E. Wluka, Flavia M. Cicuttini

**Affiliations:** a Department of Epidemiology and Preventive Medicine, School of Public Health and Preventive Medicine, Monash University, Alfred Hospital; b Baker IDI Heart and Diabetes Institute; c Mary MacKillop Institute for Health Research, Australian Catholic University, Melbourne, Australia.

**Keywords:** low back pain, physical activity, Television viewing

## Abstract

Two systematic reviews concluded that there was limited evidence to support an association between physical activity and sedentary behavior and developing low back pain (LBP). The aim of this study was to examine the associations of physical activity and television viewing time with LBP intensity and disability in community-based adults.

Five thousand fifty-eight participants (44% men) of the Australian Diabetes, Obesity and Lifestyle Study had physical activity and television viewing time measured in 1999 to 2000, 2004 to 2005, and 2011 to 2012, and LBP intensity and disability assessed in 2013 to 2014 using the Chronic Pain Grade Questionnaire. Multinomial logistic regressions were used to estimate the odds ratio for LBP intensity and disability associated with physical activity and television viewing time. Analyses were adjusted for age, education, smoking, dietary guideline index score, body mass index, and mental component summary score. To test whether associations of physical activity or television viewing time with LBP intensity and disability were modified by sex, obesity, or age, interactions were tested using the likelihood ratio test.

As gender modified the associations between physical activity and television viewing time and LBP disability (*P* = 0.05), men and women were examined separately. A total of 81.7% men and 82.1% women had LBP. Most men (63.6%) and women (60.2%) had low intensity LBP with fewer having high intensity LBP (18.1% men, 21.5% women). Most participants had no LBP disability (74.5% men, 71.8% women) with the remainder reporting low (15.8% men, 15.3% women) or high (9.7% men, 12.9% women) LBP disability. Insufficient physical activity (<2.5 hours/week) was not associated with LBP intensity or disability. High television viewing time (≥2 hours/day) was associated with greater prevalence of LBP disability in women (low disability OR 1.35, 95% CI 1.04–1.73; high disability OR 1.29, 95% CI 1.01–1.72).

Although it needs to be confirmed in RCTs our findings suggest that targeting time spent watching television and possibly other prolonged sedentary behaviors may have the potential to reduce LBP disability in community-based adults, particularly in women.

## Abstract 2

Supplemental Digital Content is available in the text

## 1 Introduction

Low back pain (LBP) contributes the highest years lived with disability (YLDs) among 291 conditions in the Global Burden of Disease 2010 study, resulting in 83 million YLDs, an increase of 42.6% since 1990.^[1]^ One in 10 people suffer from LBP worldwide at any point in time,^[1,2]^ and 70% to 85% of people have an LBP episode at some time in their life.^[2]^ This has an enormous negative economic impact on individuals, families, communities, industries, and governments.^[2]^ Understanding the etiology and risk factors for LBP is important in reducing the significant burden of this condition. In an effort to achieve this, epidemiological studies have examined a number of factors, including demographic (age and gender), obesity, and lifestyle factors (physical activity^[3]^ and sedentary lifestyle).^[4]^ The role of lifestyle factors on LBP has become a particular area of importance as they can be modified.

A systematic review examined the relationship between physical activity and LBP in 10 studies: 3 in adults and 7 in school children. It was concluded that the relationship between physical activity and LBP was too heterogeneous to reach any conclusion.^[3]^ On a closer examination, the results of the included studies were conflicting. Some studies showed beneficial effects of physical activity,^[5–8]^ whereas others showed no effect^[9,10]^ or even a detrimental effect.^[11–13]^ A further 2 studies^[14,15]^ have reported a U-shaped relationship between physical activity and LBP. Television viewing is one of the most common leisure-time behaviors that involves prolonged sitting in the domestic setting.^[16,17]^ As most people are engaged in watching television when they are not sleeping and are at home,^[17,18]^ it reflects a broader pattern of sedentary behavior. Greater television viewing time has been associated with a number of health problems, such as obesity, the metabolic syndrome, and adverse cardiometabolic biomarker changes in adults independent of their physical activity levels.^[19–21]^ Prolonged sitting could be a risk factor for developing LBP by increasing load on the spines.^[22,23]^ However, a systematic review concluded that there was limited evidence to support an association between sedentary behavior alone and developing LBP in 2009.^[4]^ Of the 15 included studies, 3 studies examined television viewing^[24–26]^ with only 1 study examining an adult population.^[24]^ Furthermore, none of these studies examined both television viewing time and physical activity. Thus the aim of this study was to examine the associations of physical activity and television viewing time with LBP intensity and LBP disability in community-based adults.

## 2 Methods

### 2.1 Study participants

**Figure 1:**
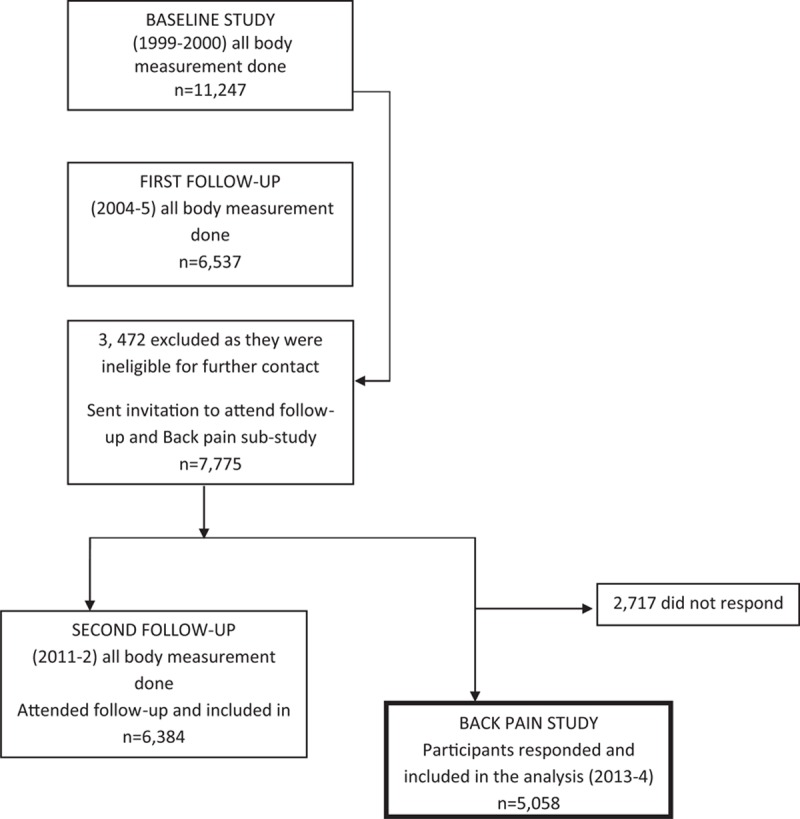
Flow diagram of recruited participants.

The Australian Diabetes, Obesity and Lifestyle (AusDiab) Study is a national, population-based cohort study of 11,247 people, of age 25 years or older, recruited by a stratified cluster sampling method during 1999 to 2000.^[27]^ AusDiab participants were followed up during 2004 to 2005 and then again in 2011 to 2012. Of the 11,247 participants, 3472 were excluded as they were ineligible for further contact (requested no further contact, deceased, too ill, or living in high-care nursing facility). In the back pain sub-study, 7775 participants were sent the back pain questionnaire between February 2013 and October 2014, of whom 5058 responded (response rate 65.1%, Fig. 1). The participants who were sent the questionnaire and who responded were younger, more educated, had a higher socioeconomic status, lower body mass index (BMI), and less television viewing time and more physical activity time than those who were not sent the questionnaire or who did not respond (Supplementary Tables 1, 2 and 3). The initial AusDiab study was approved by the International Diabetes Institute Ethics Committee and the Monash University Human Research Ethics Committee.^[27]^ The back pain substudy was approved by the Alfred Hospital Ethics Committee. All participants gave written informed consent.

### 2.2 Demographic, lifestyle factors, anthropometric and clinical measurement

Demographic and lifestyle data, including date of birth, gender, education, smoking, dietary guideline index score, were collected in 1999 to 2000 by trained interviewers using standardized questionnaires.^[27]^ The Short form 36, a self-administered questionnaire capable of producing the mental health component summary (MCS) scores was used to asses mental health quality of life.^[28]^ Height was measured to the nearest 0.5 cm without shoes using a stadiometer. Weight was measured without shoes and in light clothing to the nearest 0.1 kg using a mechanical beam balance. BMI was calculated. Blood pressure was measured with Dinamap/mercury sphygmomanometer.^[27]^ HbA1c was measured by Boronate affinity high performance liquid chromatography and serum total cholesterol measured by enzymatic method.^[27]^

### 2.3 Physical activity and television viewing time

Physical activity was assessed using the Active Australia Survey, which predominantly assesses leisure-time physical activity at baseline during 1999 to 2000, first follow-up during 2004 to 2005, and second follow-up during 2011 to 2012.^[19,29]^ Total physical activity represents the sum of time spent in walking (if continuous and 10 minutes or more), other moderate intensity activities, and vigorous intensity activity. Consistent with other international guidelines, the current Australian public health physical activity guidelines define insufficiently active if people report none or some moderate- or vigorous-intensity physical activity but <150 minutes per week, or sufficiently active if people report 150 minutes or more activity at a moderate- or vigorous-intensity level per week.^[29]^ Total time spent watching television in the previous 7 days was reported at baseline, first follow-up during 2004 to 2005, and second follow-up during 2011 to 2012.^[17]^ This did not include time when the television was switched on but other activities were being undertaken concurrently. Two categories of television viewing time (<2 and ≥2 hours/day) were created based on previously identified associations with glucose metabolism,^[19]^ retinal vascular caliber,^[30]^ and mortality.^[17]^ Both measures have shown acceptable precision: intraclass correlation coefficient 0.59, 95% confidence interval (CI) 0.52–0.65 for physical activity^[29,31]^ and intraclass correlation coefficient 0.82, 95% CI 0.75–0.87 for television viewing.^[16,30]^ These measures have provided a reliable and valid estimate of physical activity and television viewing time in adults (criterion validity 0.3, representing reasonable correlation).^[16,30]^

### 2.4 Low back pain intensity and disability

The self-administered Chronic Pain Grade Questionnaire (CPGQ) was used to assess self-reported LBP intensity and disability over the past 6 months (Supplementary material 4). The CPGQ is a reliable and valid instrument of LBP for use in population surveys.^[32–34]^ The questionnaire includes 7 questions from which a pain intensity score (0–100) and disability points score (0–6) were calculated. Based on the pain intensity score, the severity of LBP was grouped as no pain (=0), low pain intensity (<50), and high pain intensity (≥50).^[32–34]^ Similarly, based in the disability points score, LBP disability was grouped as no disability (=0), low disability (<3), and high disability (≥3).^[32–34]^

### 2.5 Statistical analysis

Multinomial logistic regression models were used to estimate the odds ratio (OR) for LBP intensity and disability associated with physical activity and television viewing time. The persistence of physical activity and television viewing time was measured between baseline and first follow-up during 2004 to 2005. Physical activity time and television viewing time were examined as dichotomous variables and coadjusted in multivariate models. Two regression models were constructed: model 1 was adjusted for age, education, smoking status, dietary guideline index score, and BMI; model 2 was further adjusted for MCS score. To test whether associations of physical activity or television viewing time with LBP intensity and disability were modified by sex, obesity, or age, interactions were fitted, and tested using the likelihood ratio test. Analysis were repeated on participants who did not have bodily pain at baseline (n = 3961). All statistical analyses were performed using Stata 13.0 SE (StataCorp LP, College Station, TX).

## 3 Results

The characteristics of the participants are presented in Table 1. As gender modified the associations between physical activity and television viewing time and disability due to LBP (*P* = 0.05), men and women were examined separately. Most men and women had low intensity LBP with a lower proportion having high intensity LBP. LBP disability was less common. Both men and women with high intensity LBP or high LBP disability were more likely to be older, less educated, current smokers, with higher BMI and lower MCS score, less physically active and watched television for longer periods. The correlation between physical activity and television viewing was negligible (*r* = −0.015, *P* = 0.28). Almost 74% of people had a similar pattern of physical activity (*r* = −0.74 *P* =  < 0.001) and 70% of people had a similar pattern of television viewing (*r* = −0.70 *P* =  < 0.001) at baseline and at first follow-up.

**Table 1:**
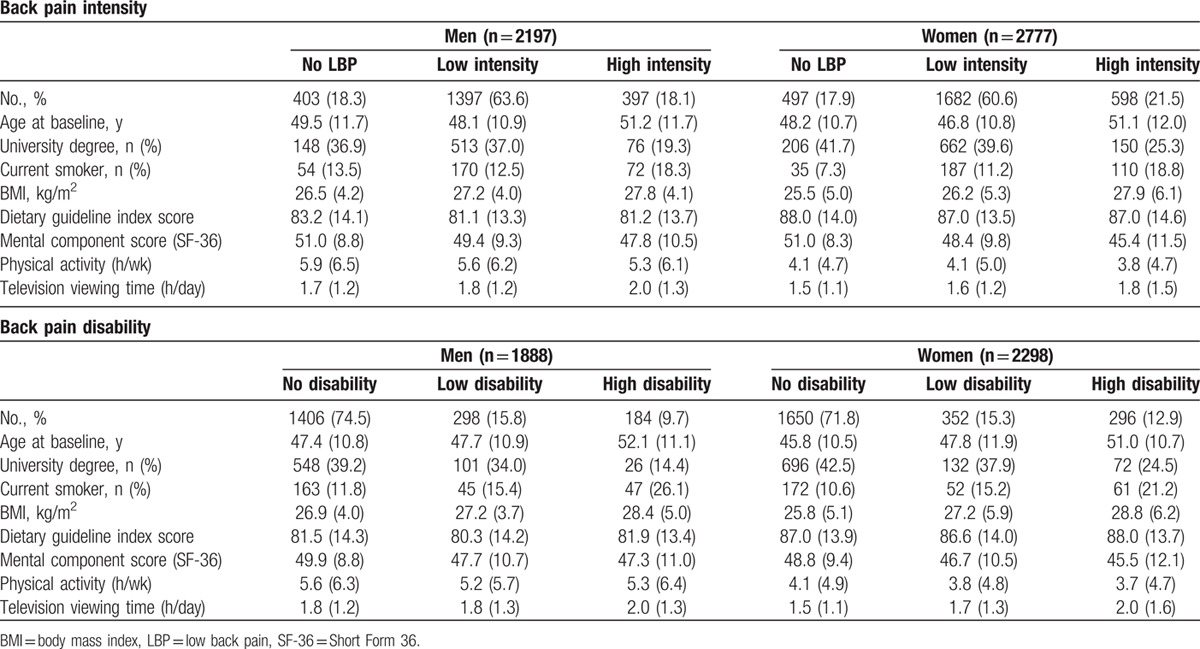
Characteristics of participants based on levels of LBP intensity and disability.

Table 2 shows the association of LBP intensity with baseline physical activity and television viewing time. After adjusted for confounders, neither physical activity nor television viewing time was significantly associated with LBP intensity, in either men or women. The association of LBP disability with physical activity and television viewing time is shown in Table 3. Neither physical activity nor television viewing time was significantly associated with low or high LBP disability in men. In women, high television viewing time was associated with increased prevalence of low LBP disability (OR 1.35, 95% CI 1.04–1.73) and high LBP disability (OR 1.29, 95% CI 1.01–1.72), with no significant association observed for physical activity. Furthermore, in this longitudinal study we performed subgroup analysis excluding those experiencing moderate to very severe bodily pain at baseline and observed similar associations (data not shown).

**Table 2:**
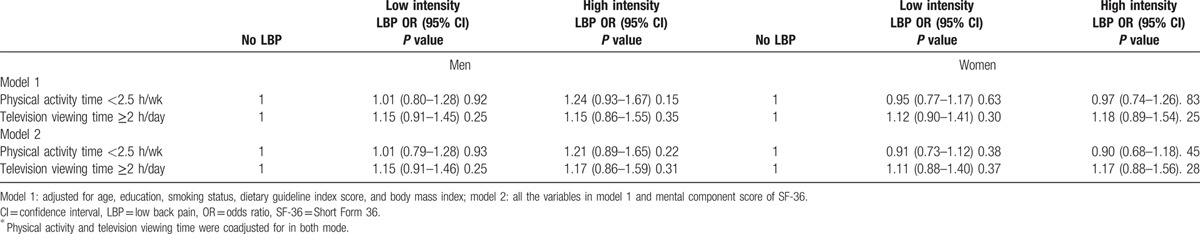
Associations of LBP intensity with physical activity and television viewing^∗^.

**Table 3:**
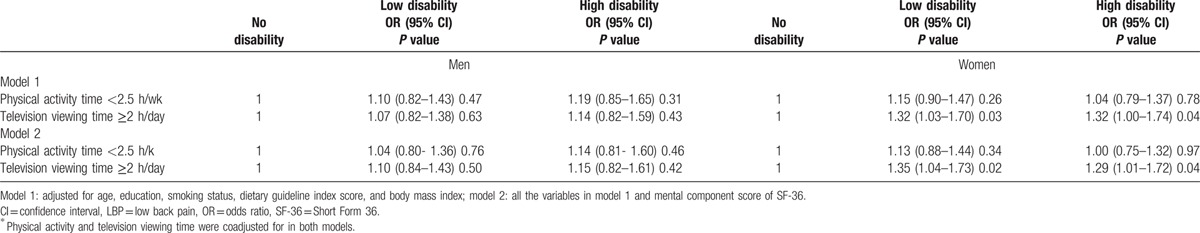
Associations of disability due to LBP with physical activity and television viewing^∗^.

## 4 Discussion

High levels of television viewing were associated with increased prevalence of disability due to LBP in women, independent of physical activity levels. No other significant associations were evident.

The evidence for the association between physical activity and LBP is contradictory as concluded by a systematic review.^[3]^ The 4 studies of adult populations included in this review were all cross-sectional in nature and reported inconsistent results.^[8,12,14,15]^ One study consisting of participants aged 18 to 23 years, 45 to 50 years, and 70 to 75 years showed that physical activity was negatively associated with LBP.^[8]^ In contrast, another study found that participants who were suffering from LBP often experienced a lower physical activity during leisure time.^[12]^ Two other studies found a U-shaped relationship between physical activity and LBP.^[14,15]^ In contrast, we found no significant association between physical activity and LBP intensity or disability. Although 2 previous studies measured LBP by asking a single question “whether the participants had back pain in the last 12 months,”^[8,12]^ our study assessed LBP intensity and disability using a valid and reliable instrument. Furthermore, our study measured physical activity approximately 15 years before the assessment of LBP.

Our study found an association between a common sedentary behavior, television viewing, and disability due to LBP in women, irrespective of their physical activity level. Our finding that higher television viewing is a risk factor for LBP is supported by other studies that have shown a higher prevalence of LBP among those whose daily activities involve prolonged sitting.^[35–38]^ Although a systematic review concluded that sedentary behavior by itself is not associated with LBP,^[4]^ only 1 study included in this review examined the relationship between television viewing and LBP in the general population,^[24]^ and the methods used to collect television viewing data and assess the severity of LBP were not adequately described.^[24]^ This is important as LBP intensity and disability differ in terms of the severity.

Data from Australia, the United Kingdom, and the United States suggest that apart from sleeping, many adults spend a large proportion of their domestic time watching television, which typically involves prolonged sitting.^[17,20]^ Our findings indicate that there is an increased risk of LBP for those who watch television for at least 2 hours per day. This finding is important from a public health perspective, because recent estimates indicate that in 2009 about 80% of households around the world owned a television,^[39]^ and the average television viewing time was approximately 3 hours in Australia and the United Kingdom and was up to 8 hours in the United States in 2007.^[17]^ In our study of a community-based population, 44% of men and 37% women watched television for at least 2 hours per day.

There are a number of potential explanations for this finding. Prolonged sitting might impact directly on lumbar spine structures and muscles. For example, previous studies showed that disc height and spine stiffness at the L4 to L5 level change after prolonged sitting without intermittent breaks.^[22,23]^ Another cross-sectional study of 72 community-based healthy volunteers showed that physical inactivity was associated with narrower lumbar intervertebral discs, high fat content of paraspinal muscles, and LBP intensity and disability.^[40]^ The association between LBP and sedentary behavior or prolonged sitting captured by television viewing might also be mediated by the effect of obesity and body composition on the lumbar spine. Several observational studies with objective measures of sedentary time that include television viewing time have reported associations of total sedentary time with body composition, obesity, and adiposity.^[17,41–44]^ It has been shown that obesity is associated with reduced disc height^[45]^ and increased fat mass is associated with LBP.^[34,46]^ In this study, however, we have adjusted for BMI. It might be argued that increased television viewing is a measure of reduced physical activity However, physical activity and television viewing are poorly correlated in the AusDiab cohort^[19]^ and in other large cohorts such as the Nurses’ Health Study.^[47]^ In our study, the association between television viewing time and LBP disability was independent of physical activity.

Although we observed an association between television viewing time and LBP disability in women, no such association was observed in men. This might be partly due to our method for assessing sitting time which focused on television viewing. With increasing use of computers, time using a computer has been included in more recent studies. In our study, information on television viewing was collected in 1999 to 2000 when use of computers was not as widespread as television viewing in this age group. There is also no evidence for a significant difference in factors associated with sedentary behavior between men and women of this age. Gender differences have been noted in previous AusDiab research for the association of television viewing time with cardiometabolic biomarkers^[19]^ and retinal venular caliber.^[30]^ For the cardiometabolic biomarkers, the relationship was stronger in women than in men,^[19]^ whereas for retinal vascular caliber, a positive association was observed in men but not in women.^[30]^ Consistent with previous studies,^[19,30]^ our study showed that men were more physically active and watched slightly more television than women. The gender differences in the association of television viewing and LBP we observed are consistent with the finding that a high amount of sitting was associated with “consultation for LBP” and “reporting LBP” in girls, but not in boys.^[11]^ Similarly another study showed a gender difference in pain levels, with women suffering from higher levels of LBP than men.^[48]^ The gender difference may be due to difference in back structure in men and women including muscle distribution, pelvic posture, and lumber spine.^[49,50]^ Recent research has shown a gender difference in postural alignments, specifically when examining back muscles, spine and pelvis postures, during prolonged sitting.^[49]^ This postural difference exposes men and women to different loading patterns that may lead to different gender-specific injury pathways and pain.^[49]^

There are several limitations. A single sedentary behavior, television viewing, was assessed in our study, although it has been shown to be a reasonable proxy measure of the overall sedentary behavior pattern.^[18]^ Television viewing time was self-reported, and no data on other sitting activities, such as working on computer, were collected. However, the information on television viewing was collected in 1999 to 2000 when leisure-time use of computers was not very prevalent. This may have led to nondifferential misclassification of prolonged sitting and is likely to have resulted in an underestimation of the strength of associations. Furthermore, participants who responded to the chronic pain grade questionnaire had better health than those who did not respond to the questionnaire. Although the cohort in the current study may not be generalizable to the whole population of Australia, these results are generalizable to a younger and healthier population. This is of significance as these people are active and in the workforce. It is possible for residual confounding that other unmeasured or unknown factors may have accounted for the associations. Reverse causality, whereby suffering from LBP at study induction may have been responsible for elevated television viewing time, cannot be ruled out. However, television viewing time and physical activity was measured during 1999 to 2000 and LBP was measured in 2013 to 2014. Strengths of our study include the recruitment of participants generally representative of Australian population, the large sample size and wide age range of the cohort, and use of a validated measure of LBP intensity and disability.

Our findings indicate that high levels of television viewing, a marker of sedentary behavior, is associated with an increased risk of LBP disability in women but not in men. Insufficient physical activity was not associated with LBP intensity or disability in either men or women. Although it needs to be confirmed in RCTs, our findings suggest that time spent watching television and possibly other prolonged sedentary behaviors including sitting in front of computers should be targeted to prevent LBP disability, particularly in women.

## Supplementary Material

Supplemental Digital Content

## Supplementary Material

Supplemental Digital Content
