# Supplementary material for: Associations between television viewing and physical activity and low back pain in community-based adults: A cohort study
Source: Medicine (Baltimore). 2016 Jun 24;95(25):e3963. doi: 10.1097/MD.0000000000003963 (PMC4998334; doi:10.1097/MD.0000000000003963)
Supplement: Supplemental Digital Content [file medi-95-e3963-s001.doc]

Supplementary table 1: Baseline characteristics of the participants who have responded and not responded in the back pain sub-study

|  | **Responded**  **n=5,058** | **Not responded**  **n=6,173** | **P** |
| --- | --- | --- | --- |
| Age, years | 48.5 (11.3) | 53.9 (16.2) | <0.001 |
| Women, n (%) | 2,819 (55.9) | 3,362 (54.5) | .15 |
| University degree, n (%) | 1,774 (35.4) | 1,453 (23.9) | <0.001 |
| Current smoker, n (%) | 632 (12.7) | 1,110 (18.4) | <0.001 |
| Socio-Economic Indexes for Area (in lowest tertile %) | 1,593 (32.1) | 2,405 (39.48) | <0.001 |
| BMI, kg/m^2^ | 26.7 (4.5) | 27.2 (5.1) | <0.001 |
| Physical activity | 4.7 (0.6) | 4.4 (0.7) | .01 |
| Television viewing time | 1.7 (1.2) | 2.0 (1.2) | <0.001 |
| Mental component score (sf-36) | 48.7 (9.8) | 48.4 (10.1) | .17 |

Supplementary table 2: Baseline characteristics comparing participants who attended the baseline visit and were invited to attend the back pain sub-study and were sent questionnaire, with those who were not sent invitation for the back pain sub-study (total n = 11,247)

|  | **Questionnaire sent**  **n=7775** | **No questionnaire sent**  **n=3472** | **P** |
| --- | --- | --- | --- |
| Age, years | 48.8 (12.3) | 57.5 (16.9) | <.001 |
| Women, n (%) | 4,322 (55.7) | 1,859 (53.8) | .06 |
| University degree, n (%) | 2,499 (32.4) | 728 (21.4) | <.001 |
| Current smoker, n (%) | 1,076 (14.1) | 666 (19.7) | <.001 |
| Socio-Economic Indexes for Area (in lowest tertile %) | 2,591 (33.9) | 1,407 (41.3) | <.001 |
| BMI, kg/m^2^ | 26.8 (4.9) | 27.3 (5.1) | <.001 |
| Physical activity | 4.7 (0.08) | 4.3 (0.07) | .01 |
| Television viewing time | 1.76 (0.02) | 2.02 (0.02) | <.001 |
| Mental component score (sf-36) | 48.7 (9.8) | 48.3 (10.4) | .17 |

Supplementary table 3: Comparison of the baseline characteristics of the participants who were send the back pain questionnaire and completed it with those who were sent the back pain questionnaire but did not respond (total n = 7775)

|  | **Responded**  **n=5058** | **Not responded**  **n=2717** | **P** |
| --- | --- | --- | --- |
| Age, years | 48.5 (11.3) | 49.5 (14.0) | .001 |
| Women, n (%) | 2,807 (55.9) | 1,515 (55.3) | .27 |
| University degree, n (%) | 1,768 (35.5) | 731 (27.9) | <.001 |
| Current smoker, n (%) | 629 (12.7) | 447 (16.6) | .001 |
| Socio-Economic Indexes for Areas (in lowest tertile %) | 1,585 (32.1) | 1,006 (37.1) | <.001 |
| BMI, kg/m^2^ | 26.7 (4.5) | 27.0 (5.0) | .04 |
| Physical activity | 4.7 (0.6) | 4.6 (0.4) | .79 |
| Television viewing time | 1.7 (1.2) | 1.8 (1.3) | <.001 |
| Mental component score (sf-36) | 48.7 (9.8) | 48.7 (9.9) | .92 |
