# Supplementary material for: Associations between television viewing and physical activity and low back pain in community-based adults: A cohort study
Source: Medicine (Baltimore). 2016 Jun 24;95(25):e3963. doi: 10.1097/MD.0000000000003963 (PMC4998334; doi:10.1097/MD.0000000000003963)
Supplement: Supplemental Digital Content [file medi-95-e3963-s002.pdf]

26821

LOW BACK PAIN

How to complete this form:

Please complete this form carefully using black ballpoint pen (not felt). Alternatively use blue pen.

Most questions only require you to answer by marking the appropriate box or boxes with a Cross like this: ☒

Please do not mark any areas outside the box.

Other questions will require a numeric answer and can be filled in like this: | 1 | 2 | 3 |

If you make a mistake when writing, cross it out with one thick line and write your correct answer above the box.

1 2 3
~~| 1 | 2 | 3 |~~

If you make a mistake, place a diagonal line through the incorrect answer like this: and then put a cross in the appropriate box of your preferred response.

~~☒~~ Yes ☒ No

Please do not cross the number 7 (eg. ~~7~~). Please make sure to write only one number in each space provided, as demonstrated in the example above.

We are interested in learning about the health of your lower back/spine.

We have defined low back pain as discomfort or pain occurring in the boxed area shown on the body diagram below.
Please refer to this when answering each of the following questions.

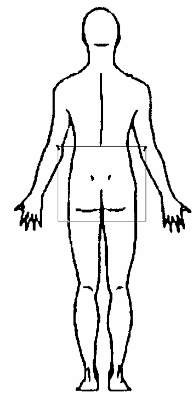

Please put a cross in one box for each question ☒

Q 1. Have you ever experienced low back pain? ☐ Yes ☐ No

Q 2. Have you had back pain (please complete each of the following (a to f)):

	Yes	No
**a)** in the past 24 hours?	☐	☐
**b)** in the past 2 weeks?	☐	☐
**c)** in the past month?	☐	☐
**d)** in the past 6 months?	☐	☐
**e)** in the past 12 months?	☐	☐
**f)** during your lifetime?	☐	☐

Q 3. Do you have low back pain now? ☐ Yes ☐ No

26821

PAIN INTENSITY

We are interested to know more about the intensity of your back pain.

The following questionnaire is the **Chronic Back Pain Grade Questionnaire** which assesses pain intensity.

For the following questions with a scale of 0-10, please place a cross in **ONE** box only

Please complete this questionnaire even if you do NOT experience back pain.

Q 4.

A. How would you rate your back pain on a 0-10 scale at the present time, which is right now, where 0 is 'no pain' and 10 is 'pain as bad as could be'?

No Pain — Pain as bad as could be

☐ 0 ☐ 1 ☐ 2 ☐ 3 ☐ 4 ☐ 5 ☐ 6 ☐ 7 ☐ 8 ☐ 9 ☐ 10

B. In the past 6 months, how intense was your worst pain rated on a 0-10 scale where 0 is 'no pain' and 10 is 'pain as bad as could be'?

No Pain — Pain as bad as could be

☐ 0 ☐ 1 ☐ 2 ☐ 3 ☐ 4 ☐ 5 ☐ 6 ☐ 7 ☐ 8 ☐ 9 ☐ 10

C. In the past 6 months, on the average, how intense was your pain rated on a 0-10 scale where 0 is 'no pain' and 10 is 'pain as bad as could be'? (That is, your usual pain at times you were experiencing pain.)

No Pain. — Pain as bad as could be

☐ 0 ☐ 1 ☐ 2 ☐ 3 ☐ 4 ☐ 5 ☐ 6 ☐ 7 ☐ 8 ☐ 9 ☐ 10

D. About how many days in the last 6 months have you been kept from your usual activities (work, school or housework) because of back pain?

☐☐☐ Disability Days

E. In the past 6 months, how much has back pain interfered with your daily activities rated on a 0-10 scale where 0 is 'no interference' and 10 is 'unable to carry on any activities'?

Interference — Unable to carry on any activities

☐ 0 ☐ 1 ☐ 2 ☐ 3 ☐ 4 ☐ 5 ☐ 6 ☐ 7 ☐ 8 ☐ 9 ☐ 10

F. In the past 6 months, how much has back pain changed your ability to take part in recreational, social and family activities where 0 is 'no change' and 10 is 'extreme change'?

No Change — Extreme change

☐ 0 ☐ 1 ☐ 2 ☐ 3 ☐ 4 ☐ 5 ☐ 6 ☐ 7 ☐ 8 ☐ 9 ☐ 10

G. In the past 6 months, how much has back pain changed your ability to work (including housework) where 0 is 'no change' and 10 is 'extreme change'?

No Change — Extreme change

☐ 0 ☐ 1 ☐ 2 ☐ 3 ☐ 4 ☐ 5 ☐ 6 ☐ 7 ☐ 8 ☐ 9 ☐ 10
